# Exploring the effects of cell seeding density on the differentiation of human pluripotent stem cells to brain microvascular endothelial cells

**DOI:** 10.1186/s12987-015-0007-9

**Published:** 2015-05-21

**Authors:** Hannah K Wilson, Scott G Canfield, Michael K Hjortness, Sean P Palecek, Eric V Shusta

**Affiliations:** Department of Chemical and Biological Engineering, University of Wisconsin-Madison, 1415 Engineering Drive, Madison, WI 53706 USA

**Keywords:** In vitro human blood–brain barrier (BBB) model, Human pluripotent stem cells (hPSCs), Brain microvascular endothelial cells, Transendothelial electrical resistance, Efflux transporters, Tight junctions, Seeding density

## Abstract

**Background:**

Brain microvascular-like endothelial cells (BMECs) derived from human pluripotent stem cells (hPSCs) have significant promise as tools for drug screening and studying the structure and function of the BBB in health and disease. The density of hPSCs is a key factor in regulating cell fate and yield during differentiation. Prior reports of hPSC differentiation to BMECs have seeded hPSCs in aggregates, leading to non-uniform cell densities that may result in differentiation heterogeneity. Here we report a singularized-cell seeding approach compatible with hPSC-derived BMEC differentiation protocols and evaluate the effects of initial hPSC seeding density on the subsequent differentiation, yield, and blood–brain barrier (BBB) phenotype.

**Methods:**

A range of densities of hPSCs was seeded and differentiated, with the resultant endothelial cell yield quantified via VE-cadherin flow cytometry. Barrier phenotype of purified hPSC-derived BMECs was measured via transendothelial electrical resistance (TEER), and purification protocols were subsequently optimized to maximize TEER. Expression of characteristic vascular markers, tight junction proteins, and transporters was confirmed by immunocytochemistry and quantified by flow cytometry. P-glycoprotein and MRP-family transporter activity was assessed by intracellular accumulation assay.

**Results:**

The initial hPSC seeding density of approximately 30,000 cells/cm^2^ served to maximize the yield of VE-cadherin+ BMECs per input hPSC. BMECs displayed the highest TEER (>2,000 Ω × cm^2^) within this same range of initial seeding densities, although optimization of the BMEC purification method could minimize the seeding density dependence for some lines. Localization and expression levels of tight junction proteins as well as efflux transporter activity were largely independent of hPSC seeding density. Finally, the utility of the singularized-cell seeding approach was demonstrated by scaling the differentiation and purification process down from 6-well to 96-well culture without impacting BBB phenotype.

**Conclusions:**

Given the yield and barrier dependence on initial seeding density, the singularized-cell seeding approach reported here should enhance the reproducibility and scalability of hPSC-derived BBB models, particularly for the application to new pluripotent stem cell lines.

**Electronic supplementary material:**

The online version of this article (doi:10.1186/s12987-015-0007-9) contains supplementary material, which is available to authorized users.

## Background

The blood–brain barrier (BBB) is composed of specialized brain microvascular endothelial cells (BMECs) that tightly control the exchange of material into and out of the brain. While necessary for maintaining normal brain function, the BBB presents a major obstacle for brain drug delivery, as it prevents most small-molecule pharmaceuticals and biologics from efficiently entering the brain [1]. At the same time, BBB dysfunction has been implicated in a variety of neurological disorders [2]. Thus, there is significant interest in studying the physiology and pathology of the BBB. Human in vitro BBB models can facilitate the detailed mechanistic studies of the BBB that would otherwise be impossible to perform in vivo, such as those involving BBB development [3–5], transport [6, 7], and disease states [8–10].

Recently, stem cells have emerged as an attractive source for constructing human in vitro BBB models. Stem cells are defined by their ability to differentiate into specialized cell types and their extensive capacity for self-renewal. Hematopoietic stem cells derived from cord blood were shown recently to differentiate into endothelial cells (ECs) and acquire BBB properties through co-culture with pericytes [11]. The resultant brain-like ECs possessed tight junctions and P-glycoprotein efflux, and displayed intermediate TEER (175 Ω × cm^2^) and paracellular permeability [11]. In a separate study, cord blood-derived circulating endothelial progenitor cells were isolated and co-cultured with astrocytes to induce BBB properties, but a substantial barrier phenotype was not demonstrated [12]. Distinct from such lineage-restricted stem cell populations, human pluripotent stem cells (hPSCs) can in principle be differentiated into any somatic cell type and exhibit unlimited expansion potential [13]. We recently published a method to differentiate hPSCs into ECs that possess many key BBB attributes (hPSC-derived BMECs), including tight junctions and expression of nutrient and efflux transporters [14]. We further showed that addition of retinoic acid (RA) during the differentiation process could significantly increase TEER to nearly 3,000 Ω × cm^2^ in monoculture [15], approaching in vivo values [16].

Importantly, the density of hPSC cultures during the differentiation process can significantly impact the differentiation yield and the resultant differentiated cell phenotype. During differentiation, hPSC density can affect the strength of paracrine signaling and the degree of cell–cell contact, both of which can regulate differentiation fates [17]. Indeed, the effect of initial hPSC seeding density on differentiation output has been noted in a number of stem cell differentiation protocols [18–20]. For example, increasing the initial hPSC seeding density in a keratinocyte differentiation protocol increased the number of K18+/p63+ simple epithelial cells, with an optimum density maximizing yield and purity [19]. In addition, initial hPSC seeding density affected the ratio of neural crest to central nervous system (CNS) neural cells in a neural differentiation study, with low densities favoring Pax6− neural crest cells and high densities favoring Pax6+ CNS neural cells [18]. Finally, another neural differentiation study found that a low initial hPSC seeding density resulted in a qualitative decrease in neural rosette formation with a slight decrease in the purity of Pax6+ neural cells [20].

Given the potential importance of initial hPSC seeding density on differentiation yield and cell properties, we developed a singularized-cell seeding approach that allows fine, uniform control of initial hPSC seeding density. Using this approach, we examined the effect of hPSC density on the resultant BMEC differentiation efficiency (% BMEC), yield (BMEC/input hPSC), and BBB phenotype. The hPSC-derived BMEC purification process was also optimized by exploring the passaging method and subculture seeding density. After defining an optimum hPSC starting density translatable to multiple hPSC lines, it was also possible to scale down the differentiation process from traditional 6-well to 96-well culture without impacting BBB phenotype, enabling a host of additional potential applications.

## Methods

### Cell culture and differentiation

IMR90-4 induced pluripotent stem cells (iPSCs), H9 human embryonic stem cells (hESCs), and DF-19-9-11T iPSCs were maintained in feeder-free conditions on Matrigel (BD Biosciences) in mTeSR1 media (WiCell Research Institute). All experiments were performed using hPSCs between passages 36–70. For routine hPSC maintenance, hPSCs were passaged with Versene (Life Technologies) every 3–5 days. For passaging hPSCs for differentiation, hPSCs were dissociated with cold Accutase (Innovative Cell Technologies) for 7 min, and the number of live cells was quantified via a hemacytometer using a trypan blue stain (Life Technologies). Singularized hPSCs were seeded with 10 μM Y27632 (ROCK inhibitor; Tocris Bioscience) for the first 24 h to promote cell attachment. After 24 h, ROCK inhibitor was withdrawn and cells were re-fed mTeSR1 every 24 h for 2 additional days. Immediately prior to initiating differentiation, the hPSC cell density of a single well was quantified via a hemacytometer to determine the day 0 hPSC starting density. To initiate differentiation, the remaining wells were switched to unconditioned media (UM): DMEM/F12 (Life Technologies) with 20% Knock Out Serum Replacement (Life Technologies), 1× MEM non-essential amino acids (Life Technologies), 1 mM GlutaMAX (Life Technologies), and 0.1 mM β-mercaptoethanol (Sigma). Cells were re-fed UM every 24 h for 6 days, followed by 2 days in endothelial cell (EC) media: hESFM (Life Technologies) with 1% platelet poor plasma-derived serum (PDS; Fisher Scientific) and 20 ng/mL bFGF (Waisman Biomanufacturing), with or without 10 μM retinoic acid (RA; Sigma) diluted in DMSO (Sigma). The final concentration of DMSO in the RA-containing EC media was 0.1%. Medium was not changed during EC phase. Unless otherwise noted, all experiments were performed using RA treatment. Following 2 days of EC treatment, BMECs were purified via selective adhesion to a collagen IV/fibronectin matrix. For seeding BMECs onto Transwells, 0.4 μm polyester Transwells (Costar) were coated with a solution of 400 μg/mL collagen IV (Sigma) and 100 μg/mL fibronectin (Sigma) in double deionized water at 37°C for at least 4 h, up to overnight. Coating solution was then aspirated, and the Transwells were allowed to dry at room temperature for at least 20 min. For seeding BMECs onto plates, tissue culture-treated polystyrene plates (Costar) were coated with a solution of 80 μg/mL collagen IV and 20 μg/mL fibronectin for at least 1 h, up to overnight. Coating solution was aspirated, and plates were allowed to dry at room temperature for at least 5 min. For seeding BMECs onto Transwells, differentiated cells were subcultured using either Versene (non-optimized method) or Accutase (optimized method). For Versene subculture onto Transwells, treatment length was kept constant at 15 min at 37°C, and the dissociated cells were seeded onto Transwells based on a constant ratio, where one 6-well of differentiated cells was split into three 12-well Transwells. For Accutase subculture onto Transwells, treatment length varied depending on differentiation state of the cells (20–25 min for cells without RA treatment, 30–45 min for cells with RA treatment, until a singularized cell suspension was formed). Accutase-dissociated cells were quantified via a hemacytometer with trypan blue staining and seeded onto Transwells at various densities. Cell viability after Accutase treatment was >90%. For seeding BMECs onto plates, Accutase subculturing was used exclusively. For both plates and Transwells, cells were subcultured into EC media with or without RA, and after 24 h culture medium was changed to hESFM with 1% PDS (withdrawing the bFGF and RA). Medium was not changed thereafter. TEER was measured every 24 h via EVOM voltohmeter with STX2 electrodes (World Precision Instruments), and all measurements were performed at 37°C to prevent fluctuations in TEER value due to temperature change. The resistance value of an empty filter coated with collagen IV and fibronectin was subtracted from each measurement.

### Permeability coefficient measurement

Sodium fluorescein (10 μM, Sigma) was diluted in hESFM + 1% PDS, and 0.5 mL was added to the upper chamber of a 12-well Transwell filter. Aliquots (200 μL) were extracted from the basolateral chamber (1.5 mL) every 15 min over the course of 1 h and replaced by an equal volume of fresh medium. The rates of accumulation in the basolateral chamber, as well as that across an empty filter coated with collagen IV and fibronectin, were used to calculate the P_e_ value. During measurement, filters were incubated at 37°C on a rotating platform.

### Immunocytochemistry

Cells were fixed in either 100% ice-cold methanol or 4% paraformaldehyde in PBS for 15 min at room temperature, washed three times with PBS (Sigma), and blocked for 30 min with either 10 or 40% goat serum (Sigma) in PBS. Cells were incubated with primary antibody diluted in 10% or 40% goat serum overnight at 4°C on a rocking platform (see Additional file 1: Table S1). Cells were washed three times with PBS and incubated with goat anti-mouse or anti-rabbit Alexa Fluor 488 (1:200; Life Technologies) in 10 or 40% goat serum. For co-staining of nestin and VE-cadherin, mouse isotype-specific antibodies were used (1:200; Life Technologies). Cell nuclei were labeled with 4′,6-Diamidino-2-pheny-lindoldihydrochloride (DAPI; Sigma) for 10 min. Cells were washed three times with PBS and visualized.

### Flow cytometry

VE-cadherin flow cytometry was performed on live cells, and all incubation steps were performed at room temperature on an orbital shaker. First, live cells were blocked for 30 min in 40% goat serum in PBS, and then incubated with primary antibody diluted in 40% goat serum for 1 h (see Additional file 1: Table S1). Cells were then washed three times with PBS and incubated with goat anti-mouse Alexa Fluor 488 (Life Technologies, 1:200 dilution) in 40% goat serum for 30 min. Cells were dissociated with Versene for 1 h, triturated vigorously, passed through a 40 μm mesh filter (BD Falcon), and post-fixed with 100% ice-cold methanol for 15 min. Cells were washed twice in PBS containing 1% BSA (Sigma) and analyzed on a FACSCalibur flow cytometer. For all other antigens, cells were dissociated via Accutase or Versene for 15–30 min, fixed for 15 min with 2% paraformaldehyde or ice-cold methanol, and washed twice with PBS containing 1% BSA. Next, cells were blocked with 10% donkey serum (Sigma) or 10% goat serum in PBS for 30 min, with or without 0.1% Triton-X (Sigma) for permeabilization, and then incubated with primary antibody solution in 10% donkey serum or 10% goat serum overnight at 4°C on a rotating platform. The next day, cells were washed twice with PBS containing 1% BSA, incubated for 30 min with goat anti-mouse Alexa Fluor 488 (1:200) in 10% donkey or 10% goat serum, and again washed twice in PBS containing 1% BSA. Cells were analyzed on FACSCalibur flow cytometer.

### Efflux transporter activity

Efflux transporter activity was assessed via intracellular accumulation of fluorescent transporter substrates. Purified BMECs were pre-incubated with or without specific inhibitors, either 100 μM MK571 (Sigma) or 10 μM cyclosporin A (CsA; Sigma) in HBSS buffer (Life Technologies) for 1 h at 37°C on an orbital shaker. Cells were then incubated with substrate, either 10 μM carboxymethyl-2′,7′-dichlorofluorescein diacetate (DCFDA; Life Technologies) or 10 μM rhodamine 123 (Sigma) with or without specific inhibitors, for an additional hour at 37°C on an orbital shaker. Cells were washed twice with cold PBS and lysed with RIPA buffer (Pierce Biotechnology), and fluorescence was measured on a plate reader (485 nm excitation and 530 nm emission). Fluorescence values were subsequently normalized to cell number by counting dissociated cells collected from parallel, unlysed wells via hemacytometer and reported as normalized accumulation.

### Statistical analysis

Data were expressed as mean ± standard deviation (SD). Student’s unpaired *t* test was used to determine statistical significance between groups. A value of *p* < 0.05 was considered statistically significant.

## Results

### Effect of initial hPSC seeding density on BMEC yield

**Figure 1:**
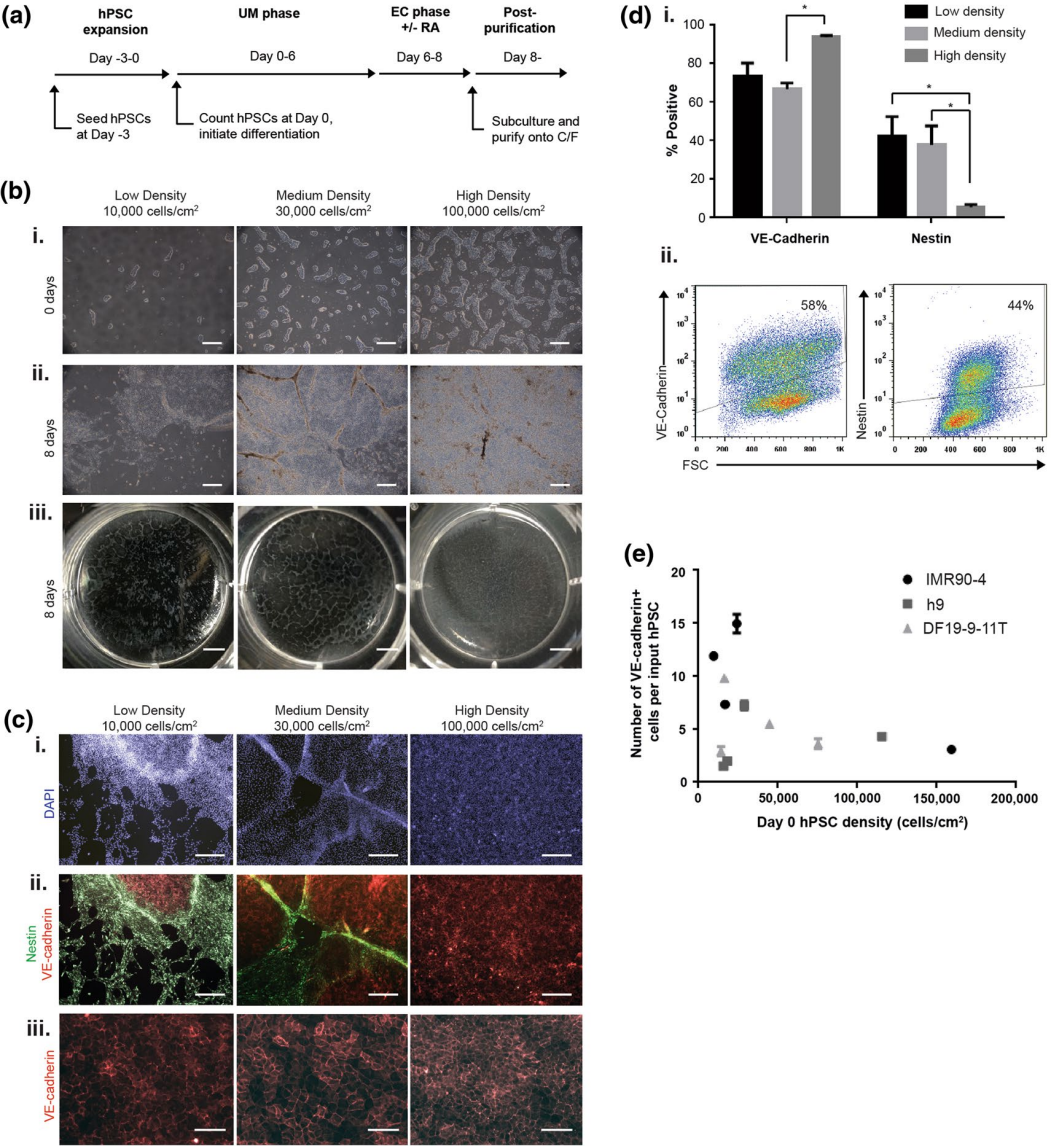
Effect of day 0 hPSC density on differentiation efficiency and yield of brain microvascular endothelial cells. **a** Schematic representation of BMEC differentiation protocol. hPSCs were seeded as singularized cells at day −3 and induced to differentiate at day 0. Cells were differentiated for 6 days in UM followed by 2 days in EC media (10 μM RA treatment was included for all reported data unless otherwise noted) and subcultured onto collagen/fibronectin matrix. *UM* unconditioned medium, *EC* endothelial cell, *RA* retinoic acid, *C/F* collagen/fibronectin. **b** Brightfield images of IMR90-4 BMEC differentiation. Recorded densities are the day 0 hPSC starting densities. *i* Brightfield images of IMR90-4 iPSCs at day 0, following 3 days of expansion in pluripotency media. *Scale bars* 500 μm. *ii* Brightfield images after 8 days differentiation. *Scale bars* 500 μm. *iii* Unmagnified images of differentiated cells in 6-well tissue-culture plate following 8 days of differentiation. *Scale bars* 5 mm. **c** *i* and *ii* Expression of VE-cadherin (*red*) and nestin (*green*) in IMR90-4 cells differentiated for 8 days. *iii* Higher magnification images of VE-cadherin+ EC colonies. *Scale bars*: *i* and *ii* 500 μm, *iii* 100 μm. **d** Quantification of VE-cadherin and nestin expression in IMR90-4 iPSCs. *i* Quantification via flow cytometry of percent VE-cadherin+ and nestin+ following 8 days of differentiation. VE-cadherin and nestin flow cytometry were compared to appropriate mouse IgG control antibody. Values are mean ± SD of three independent differentiations. Statistical significance was calculated via Student’s unpaired t test (*p < 0.05). *ii* Representative flow cytometry dot plots from medium density culture. *Gates* represent expression above mouse IgG control. **e** Quantification of VE-cadherin yield per input hPSC as a function of the day 0 hPSC starting density. IMR90-4 iPSCs were differentiated for 8 days, and the yield was calculated as the % VE-cadherin+ as measured by flow cytometry multiplied by total cell number at 8 days and normalized to the day 0 hPSC starting density. Values are the mean ± SD of two biological replicates from a single differentiation. The experiment was repeated for two independent differentiations to verify reported trends.

In previous BMEC differentiation protocols, undifferentiated hPSCs were seeded as Versene-dissociated colonies prior to initiating BMEC differentiation [14, 15]. While these approaches are effective, it is not trivial to control the colony size and maintain homogenous colony distribution during seeding. Therefore, the cell density at the start of differentiation often varied between experiments. To determine how hPSC density affects BMEC differentiation, a singularized-cell seeding approach was developed to allow for more precise control and more uniformity of hPSC density at the initiation of differentiation. IMR90-4 iPSCs were treated with Accutase to promote the formation of a singularized cell suspension, counted, and seeded onto Matrigel at various densities in the presence of ROCK inhibitor Y27632 at day −3 (Figure 1a) to promote cell attachment and survival [21]. The length of expansion in mTeSR1 media, which was previously variable between 2 and 3 days [14, 15], was set to 3 days. During the 3-day expansion phase, singularized hPSCs were observed to proliferate and form clusters of cells (Figure 1bi). At day 0, differentiation was initiated at hPSC cell densities of 10,000, 30,000, or 100,000 cells/cm^2^ (low, medium, and high density, Figure 1a, b) by switching to unconditioned medium (UM) for 6 days, followed by 2 days in EC medium supplemented with 10 μM RA (all reported data below include the presence of RA unless otherwise noted). By the 8-day time-point, substantial differences in overall cell density were observed at the macroscopic level (Figure 1b). During the BMEC differentiation process, populations of neural cells and ECs co-differentiate [14]. Accordingly, immunostaining revealed that VE-cadherin+ ECs and nestin+ immature neural cells were present at each density, but differences in the percentages of VE-cadherin+ ECs were observed (Figure 1c, d**)**. At a starting cell density of 10,000 cells/cm^2^, colonies of VE-cadherin+ cells were sparse and surrounded by low-density regions of nestin+ cells (Figure 1c). At 30,000 cells/cm^2^, greater numbers of VE-cadherin+ colonies were observed, surrounded by tightly packed tracts of nestin+ neural cells. At starting cell densities ≥100,000 cells/cm^2^, uniform VE-cadherin staining was observed, and few nestin+ cells remained at this time point. A similar trend between hPSC density and resultant nestin+ and VE-cadherin+ cell populations was noted for two additional hPSC lines, H9 hESC and DF-19-9-11T iPSC lines (Additional file 1: Figure S1).

Flow cytometric quantification revealed comparable percentages of VE-cadherin+ cells in low- vs. medium-density cultures (73 ± 7 and 67 ± 8% VE-cadherin, respectively), while in high-density cultures, the percentage of VE-cadherin+ cells increased to 94 ± 1% (Figure 1d). While the lack of co-differentiating nestin+ cells in high-density cultures is striking (only 5 ± 1% nestin+ cells at high density compared to 38 ± 10% at medium density), we did observe the increased presence of nestin+ cells in high-density cultures at an earlier time point. Evaluation of high-density cultures at 4 days UM indicated that nestin+ cells comprised 20% of the population (Additional file 1: Figure S2). Interestingly, if differentiation was initiated at 30,000 cells/cm^2^ after only 1 day of expansion in mTeSR1 instead of the standard 3 days, a decrease in VE-cadherin+ cells was observed (39 ± 23% VE-cadherin+, Table 1), suggesting that the expansion phase can play a role in determining the fraction of the co-differentiating cultures that become VE-cadherin+ ECs.

**Table 1:** Initial seeding density effects on hPSC-derived brain microvascular endothelial cells

Cell line	Days in mTeSR1	Density at D0 (cells/cm^2^)	RA conc. (μM)	% VE-cadherin+^a^	Max. TEER (Ω × cm^2^)^b^
IMR90-4	3	30,000	10	67 ± 8	2,300 ± 290
	3	100,000	10	94 ± 1	2,310 ± 100
	3	30,000	0	ND	310 ± 50
	1	30,000	10	39 ± 23	1,860 ± 470
DF19-9-11	3	10,000	10	72 ± 10	960 ± 40
	3	30,000	10	76 ± 7	670 ± 40
	3	100,000	10	92 ± 6	450 ± 100
H9	3	30,000	10	69 ± 15	2,190 ± 500
	3	100,000	10	73 ± 1	375 ± 410

*ND* not determined, *RA* retinoic acid.

^a^Percent of population that is VE-cadherin+ after 8 days differentiation as measured by flow cytometry. Values are mean ± SD of at least two independent differentiations.

^b^Maximum transendothelial electrical resistance using optimized subculture method (Accutase and subculture density of 1 million cells/cm^2^). Values are mean ± SD of at least two independent differentiations.

Although there was a significant increase in VE-cadherin+ EC percentages in high-density cultures compared to medium density, there was a clear optimum of VE-cadherin+ cell yield per input hPSC at a day 0 hPSC seeding density of 30,000 cells/cm^2^, which generated a maximum of 15.0 VE-cadherin+ ECs/input IMR90-4 iPSC at day 8 (Figure 1e). High-density culture, on the other hand, generated only 3.1 VE-cadherin+ ECs/input IMR90-4 iPSC. Interestingly, H9 and DF19-9-11 hPSCs also demonstrated an optimum yield in this same range of hPSC starting density of ~30,000 cells/cm^2^ (Figure 1e). However, the maximum yields for these lines were somewhat lower at 7.2 VE-cadherin+ cells/input H9 hESC, and 9.8 VE-cadherin+ cells/input DF-19-9-11T iPSC. Thus, for all 3 hPSC lines tested, the initial hPSC seeding density of approximately 30,000 cells/cm^2^ served to optimize the EC differentiation yield.

### Effect of initial hPSC cell seeding density on transendothelial electrical resistance

**Figure 2:**
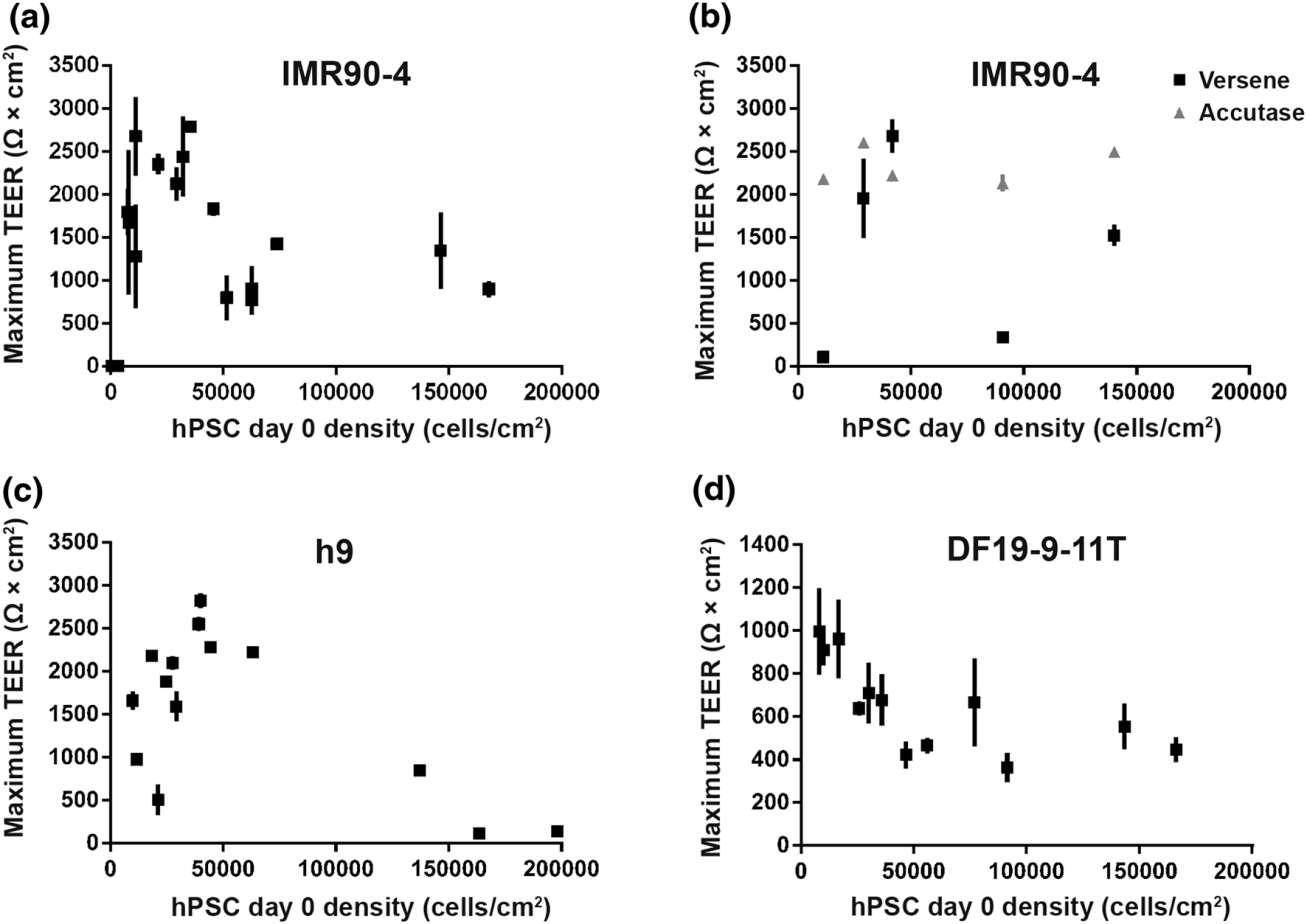
Effect of day 0 hPSC seeding density on maximum transendothelial electrical resistance of derived brain microvascular endothelial cells. **a** IMR90-4 iPSCs were differentiated for 8 days and subcultured onto Transwells using Versene. **b** IMR90-4 iPSCs were differentiated for 8 days and were either subcultured using Versene or Accutase dissociation and then seeded onto filters at density of 1 million cells/cm^2^. Versene and Accutase data are paired from the same differentiation. H9 hESCs (**c**) or DF19-9-11T iPSCs (**d**) were differentiated for 8 days and subcultured with Accutase and seeded onto filters at a density of 1 million cells/cm^2^. **a**–**d** Data were collected from between 2 to 5 independent differentiations, with each data point representing the mean ± SD of triplicate Transwells from a single differentiation.

Barrier tightness in the form of TEER was next evaluated as a function of the day 0 hPSC seeding density. At day 8 of differentiation, hPSC-derived BMECs were purified via selective adhesion to collagen IV/fibronectin-coated Transwell filters in a monoculture format using Versene subculture as previously described [15], and TEER was measured every 24 h. For the IMR90-4 iPSC line, a maximum TEER of 2,430 ± 130 Ω × cm^2^ was observed using a day 0 hPSC seeding density of 30,000 cells/cm^2^, and maximum TEER decreased significantly outside this optimum hPSC density (Figure 2a).

To determine whether the strong density dependence of TEER on cell seeding density was a result of BMEC quality or the method of purification, the effect of cell dissociation method on subculture were explored. We noted that for high-density cultures (i.e., hPSC starting density ≥100,000 cells/cm^2^), Versene dissociation tended to yield large aggregates of cells, as opposed to the near singularized-cell suspension observed for lower density cultures (e.g., ≤30,000 cells/cm^2^) having undergone the same treatment. Further, high-density cultures did not form uniform cellular monolayers on the Transwells, as large clusters of cellular debris remained attached to the surface even after medium change (Additional file 1: Figure S3). Therefore, a purification technique using prolonged Accutase treatment, rather than Versene dissociation, was developed to promote the formation of a uniform singularized cell suspension. Dissociating cells in this manner also allowed accurate quantification of the cell suspension and better control over the filter seeding density, a variable that has been previously noted to affect TEER in immortalized BMECs [22]. In order to reach the highest TEER over a 7-day period, the minimum filter seeding density of hPSC-derived BMECs was determined to be 1 million cells/cm^2^. The maximum TEER was generally observed 48 h post-purification and remained elevated above 1,000 Ω × cm^2^ for at least 7 days post-purification (Additional file 1: Figure S4).

Using this optimized subculturing method (Accutase and 1 million cells/cm^2^), the effects of day 0 hPSC seeding density on TEER were re-evaluated. We found that IMR90-4-derived BMECs could reach similarly high TEER values of ≥2,000 Ω × cm^2^ regardless of the day 0 hPSC seeding density (Figure 2b; Table 1). Further, these high TEER values were accompanied by a low sodium fluorescein permeability coefficient of 1.6 × 10^−7^ cm/s. In contrast to IMR90-4-derived BMECs, H9- and DF19-9-11T-derived BMECs still displayed diminished TEER outside the optimum day 0 hPSC seeding density even when using the optimized subculture and purification methods (Figure 2c, d). At a starting cell density in the range of 30,000–45,000 cells/cm^2^, H9-derived BMECs achieved an average maximum TEER of 2,200 ± 410 Ω × cm^2^, but the TEER dropped below 1,000 Ω × cm^2^ at day 0 seeding densities ≥100,000 cells/cm^2^ (Figure 2c; Table 1). The day 0 hPSC density that achieved the highest TEER for DF19-9-11T-derived BMECs was 10,000 cells/cm^2^ (960 ± 40 Ω × cm^2^), although 30,000 cells/cm^2^ still resulted in a TEER of 670 ± 40 Ω × cm^2^ (Figure 2d; Table 1). Similar to the results with H9-derived BMECs, the maximum TEER dropped to ~500 Ω × cm^2^ at starting cell densities ≥100,000 cells/cm^2^ (Figure 2d; Table 1). All of the aforementioned data was generated using RA treatment during the differentiation. If instead IMR90-4 BMECs were differentiated at a day 0 density of 30,000 cells/cm^2^ without RA treatment, a maximum TEER of 310 ± 50 Ω × cm^2^ was achieved (Table 1), similar to previously reported values [15].

### Effect of day 0 hPSC seeding density on BMEC phenotype

**Figure 3:**
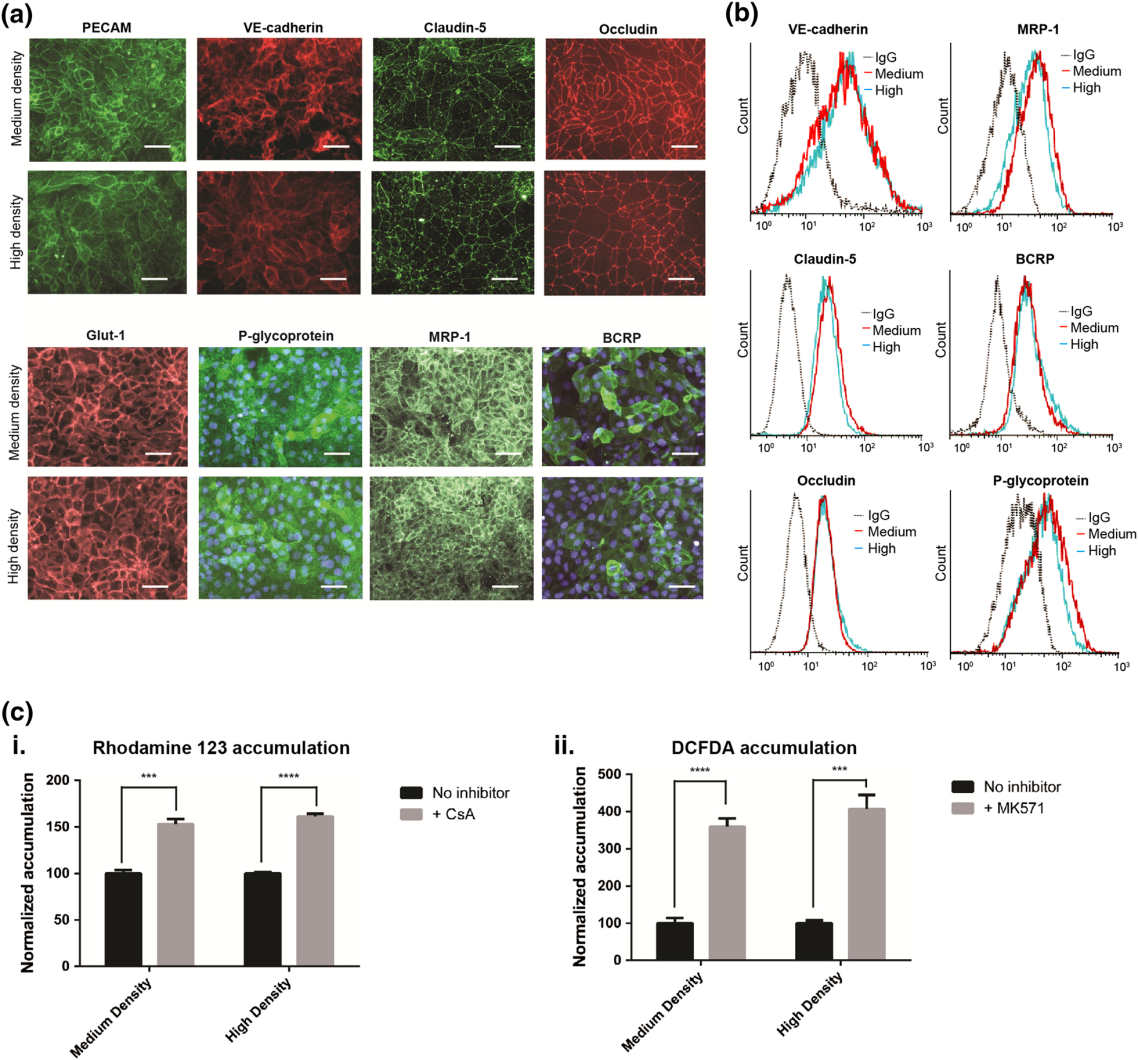
Comparison of BBB phenotypic characteristics between medium- and high-density cultures. All experiments were performed on purified IMR90-4-derived BMECs at day 10 of differentiation. **a** Immunostaining of PECAM, VE-cadherin, claudin-5, occludin, Glut-1, P-glycoprotein, MRP-1, and BCRP. DAPI overlay is included on P-glycoprotein and BCRP images. *Scale bars* 50 μm. **b** Flow cytometry histograms of VE-cadherin, claudin-5, occludin, P-glycoprotein, MRP-1, and BCRP. Appropriate mouse IgG control is included for each marker. The difference in expression as assessed by the geometric means of the labeled populations was not greater than 20% between medium- and high-density cultures for any marker in at least two independent differentiations. **c** Efflux transporter inhibition as measured via intracellular accumulation of either rhodamine 123 (*i*) or DCFDA (*ii*). Inhibitor-treated samples were independently normalized to each respective non-inhibitor-treated control sample. Statistical significance was calculated using the Student’s unpaired t test (***p ≤ 0.001, ****p ≤ 0.0001). Values are mean ± SD of three replicates from a single differentiation, and experiments were repeated for two additional independent differentiations for verification of reported trends.

Having established that IMR90-4-derived BMECs could achieve high TEER across a wide range of hPSC starting densities, the effect of day 0 hPSC density on BBB marker expression and efflux activity was next evaluated. Day 0 hPSCs seeded at either 30,000 cells/cm^2^ (“medium density”) or 100,000 cells/cm^2^ (“high density”) were differentiated and purified using the optimized subculture method. Low-density cultures were excluded from this analysis given their restrictively low BMEC yield. IMR90-4-derived BMECs generated from both medium and high day 0 hPSC seeding densities expressed standard BBB markers as assessed by immunocytochemistry and flow cytometry. Both medium- and high-density cultures expressed PECAM-1 and VE-cadherin as well as the glucose transporter GLUT-1. Similarly, claudin-5 and occludin tight junction proteins were expressed in both systems (Figure 3a). Flow cytometric analysis of the junction proteins VE-cadherin, claudin-5 and occludin indicated there were no substantial differences in expression levels between medium- and high-density-derived BMECs (Figure 3b), correlating with the indistinguishable TEERs in the optimized subculture system. Efflux transporters P-glycoprotein, breast cancer resistance protein (BCRP), and multidrug resistance protein-1 (MRP-1) were also expressed (Figure 3a), and again the medium- and high-density expression levels as assessed by flow cytometry were indistinguishable (Figure 3b). Qualitative assessment of purified H9- and DF-19-9-11T-derived BMECs also demonstrated expression of the panel of BBB markers in both medium- and high-density cultures (Additional file 1: Figure S5).

In addition to assessing the effects of day 0 hPSC seeding density on efflux transporter expression levels by flow cytometry, efflux transporter activity was also compared by accumulation assay. Intracellular accumulation of fluorescent P-glycoprotein and MRP-family substrates was measured in the presence and absence of specific inhibitors. Following treatment with P-glycoprotein inhibitor cyclosporin A, accumulation of P-glycoprotein substrate rhodamine 123 was inhibited to a similar degree in medium- versus high-density cultures (Figure 3ci). Likewise, accumulation of DCFDA, an MRP family substrate, was inhibited to a similar degree by MRP-family inhibitor MK571 between medium and high-density cultures (Figure 3cii). These findings also applied to both H9- and DF-19-9-11T-derived BMECs (Additional file 1: Figure S6). Taken together, the expression levels and activities of those BBB proteins tested were not affected within the threefold range of hPSC seeding densities.

### Effects of differentiation scale-down on BMEC phenotype

**Figure 4:**
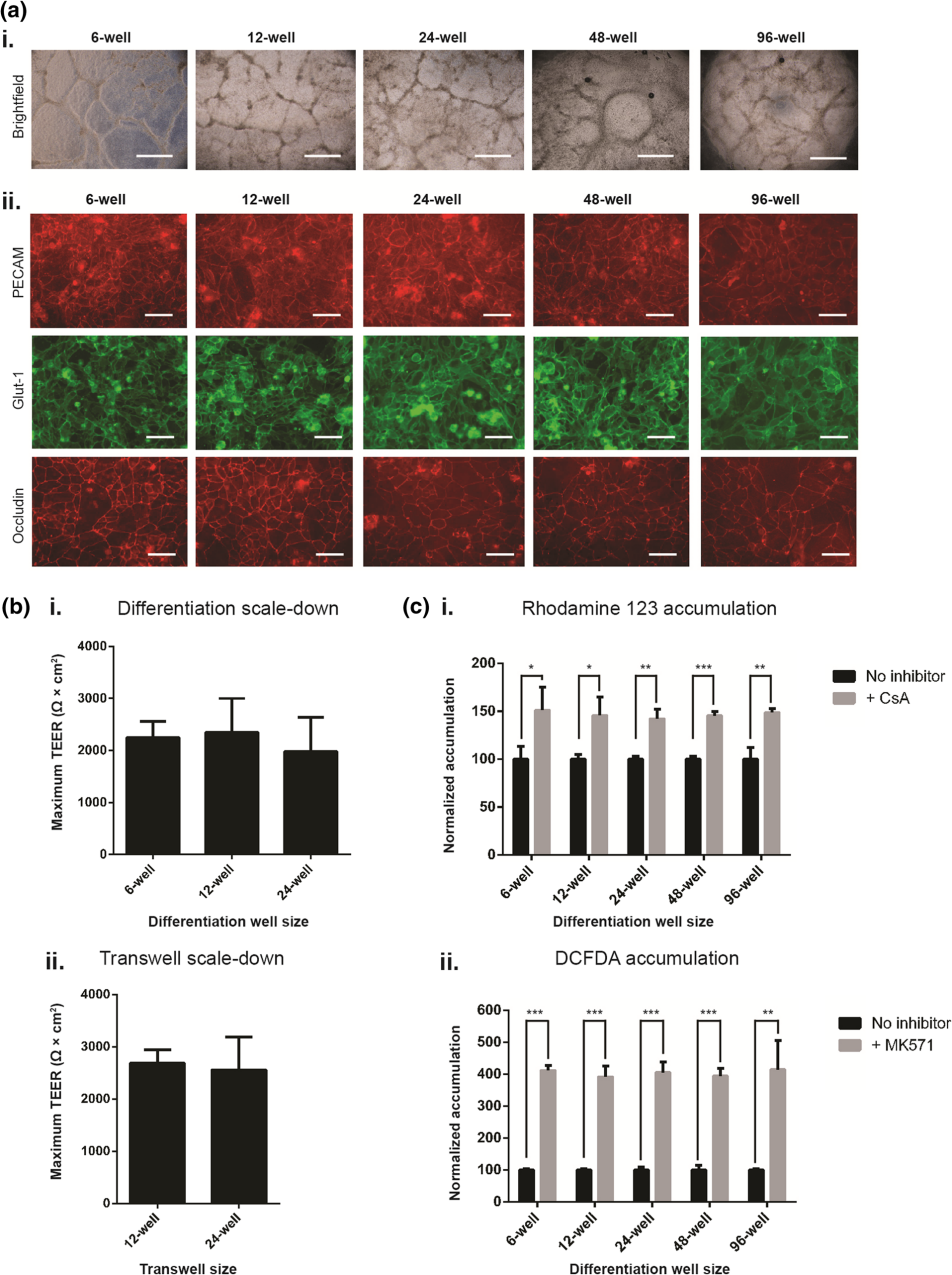
Day 0 hPSC starting density can be used to scale down brain microvascular endothelial cell differentiation from traditional 6-well culture to 12-, 24-, 48-, and 96-well cultures. **a** *i* Brightfield images of IMR90-4-derived BMECs differentiated in 6-, 12-, 24-, 48-, or 96-well format for 8 days. *Scale bars* 1 mm. *ii* Immunostaining of purified IMR90-4-derived BMECs at day 10 following differentiation in either 6-, 12-, 24-, 48-, or 96-well plate format. *Scale bars* 50 μm. **b** *i* Maximum TEER of IMR90-4-derived BMECs differentiated in either 6-, 12-, or 24-well format and purified by subculturing onto 12-well Transwells at a filter seeding density of 1 million cells/cm^2^. Data from each 6-, 12-, and 24-well differentiation were paired and averaged from four independent differentiations. *ii* Maximum TEER of IMR90-4-derived BMECs differentiated in 6-well format and subcultured onto either 12- or 24-well Transwells at a filter seeding density of 1 million cells/cm^2^. Data from 12- and 24-well Transwells were paired and averaged from two independent differentiations. **c** IMR90-4-derived BMECs were differentiated in 6-, 12-, 24-, 48-, or 96-well format, and efflux transporter inhibition of purified cultures at day 10 was measured via intracellular accumulation of *i* rhodamine 123 or *ii* DCFDA. Inhibitor-treated samples were independently normalized to each respective non-inhibitor-treated control sample. Statistical significance was calculated using Student’s unpaired t test (*p ≤ 0.05, **p ≤ 0.01, ***p ≤ 0.001). Values are mean ± SD of three replicates from a single differentiation, and experiments were repeated for two additional independent differentiations for verification of reported trends. All differentiations in panels **a**–**c** were initiated at a day 0 hPSC starting density of 30,000 cells/cm^2^.

To date, hPSCs-derived BMECs have been differentiated in 6-well plates (surface area 9.5 cm^2^) and purified via subculture onto 12-well plates (surface area 3.8 cm^2^) or 12-well Transwells (surface area 1.1 cm^2^). A potential advantage to having optimized the day 0 hPSC starting density is the capability to easily scale the differentiation system simply by adhering to the day 0 hPSC seeding density of 30,000 cells/cm^2^. Therefore, hPSCs were seeded into 6-, 12-, 24-, 48-, and 96-well plates based on the optimum hPSC starting density of 30,000 cells/cm^2^, and the differentiation was carried out as normal. At day 8, differentiating cultures across all well sizes displayed similar morphology to that seen previously in Figure 1b, with flat EC colonies surrounded by interconnected tracts of neural cells (Figure 4ai). BMECs were then purified by subculturing onto 96-well plates, and the cells were assayed for expression of selected BBB and vascular markers. Qualitative immunocytochemical analysis indicated similar PECAM-1, Glut-1, and occludin expression profiles across all well sizes (Figure 4aii), suggesting that hPSC differentiation can be scaled down to a 96-well format without substantially affecting the marker signature of hPSC-derived BMECs.

In terms of barrier properties, BMECs differentiated in 6-, 12-, or 24-well plates achieved indistinguishable maximum TEER values on 12-well filters using Accutase subculture and a filter seeding density of 1 million cells/cm^2^ (Figure 4bi). BMECs differentiated in 48- and 96-well plates, on the other hand, were not subcultured onto Transwells due to practical issues acquiring the requisite number of BMECs. Because BBB transport assays could benefit from the use of scaled-down Transwell systems, scale-down from 12-well to 24-well Transwells was evaluated. BMECs were purified by subculture on to either the standard 12-well Transwells (1.1 cm^2^) or 24-well Transwells (0.32 cm^2^) at the optimized filter seeding density of 1 million cells/cm^2^, which yielded equivalent maximum TEER values (Figure 4bii). Finally, P-glycoprotein and MRP-1 efflux transporter activity as measured by accumulation assay was indistinguishable using purified BMECs resulting from differentiation in 6-, 12-, 24-, 48-, and 96-well plates (Figure 4ci, ii). Overall, employing the optimized day 0 hPSC seeding density and subculture passaging techniques allows for effective scaling of the hPSC–BMEC differentiation system.

## Discussion

hPSC differentiation protocols that are well-defined, reproducible, and scalable can be highly beneficial, particularly for high throughput drug screens requiring large numbers of cells that exhibit low batch-to-batch variability [23]. We previously reported that hPSCs could be differentiated into ECs possessing key BBB attributes [14] and further, that RA treatment could enhance many of these properties, most strikingly the barrier tightness [15]. In this study we sought to develop more standardized methods of generating hPSC-derived BMECs in order to increase differentiation reproducibility and scalability. To this end, a singularized-cell seeding protocol was established to control more precisely the starting hPSC density, leading to the identification of an optimum day 0 seeding density of 30,000 cells/cm^2^ that served to maximize yield and barrier tightness for three different hPSC lines. Optimized subculturing approaches mitigated the day 0 effect on barrier tightness for the IMR90-4 iPSC line. Accordingly, purified IMR90-4 BMECs having similarly high TEER values derived from day 0 seeding densities of 30,000 and 100,000 cells/cm^2^ possessed similar amounts and localization of adherens and tight junction proteins. Efflux transporter expression and activity also were unchanged over this range of day 0 seeding densities. Finally, optimized day 0 seeding density also allowed facile scale-down of the differentiation process. Taken together, it is predicted that the differentiation and subculturing protocols described here will allow translation of hPSC differentiation to BMECs to many other laboratories.

As mentioned above, day 0 hPSC seeding density significantly affected BMEC percentage and yield, with higher hPSC seeding densities promoting higher percentages of VE-cadherin+ ECs in the co-differentiating mixture. An IMR90-4 iPSC seeding density of 30,000 cells/cm^2^ (“medium density”) differentiated for 8 days generated a mixture of 67 ± 8% VE-cadherin+ ECs. We previously reported BMEC purities of 66 and 67% in non-RA and RA treated cultures, respectively [14, 15], nearly identical values to those reported here for the medium-density condition. Medium density also maximized BMEC yield per input hPSC, with IMR90-4-derived BMECs displaying a yield of 15.0 VE-cadherin+ ECs per input iPSC. This value was slightly higher than the 11.6 BMECs per input iPSC reported previously [14]. Therefore, the singularized-cell seeding approach at medium density generates similar purities and yields as previous model iterations and does so in a more controllable manner. At higher day 0 seeding densities of 100,000 cells/cm^2^, the day 8 differentiating mixture was 94 ± 1% VE-cadherin+ ECs. The higher EC percentage correlated with a lower number of nestin+ cells at the 8-day time point (5 ± 1% nestin+ neural cells at high density compared to 38 ± 10% at medium density). Given the importance of the co-differentiating neural population in the resultant BMEC cells, for example through Wnt pathway modulation [14], and the relatively unchanged BMEC phenotype in the high density cultures, neural cell presence was investigated earlier in the differentiation. These experiments indicated that at 4 days of UM treatment in the high-density cultures there existed a significant nestin+ neural cell component comprising 20% of the population. Therefore, high-density culture may alter the timing but apparently does not remove the potential positive impact of the co-differentiating nestin+ neural cells, given the largely unaltered BMEC phenotype after purification.

We also developed an approach for purifying hPSC-derived BMECs via subculture onto Transwells by optimizing the filter seeding density, which has been noted previously as an important variable for maximizing TEER [22]. Prolonged Accutase treatment was used to generate singularized suspensions of differentiated cells, and the optimum Transwell seeding density was found to be 1 million cells/cm^2^. Using the standardized filter seeding approach, IMR90-4-derived BMECs at medium density had a maximum TEER of 2,300 ± 290 Ω × cm^2^ following RA treatment. Previously, RA-treated IMR90-4-derived BMECs were reported to reach a maximum TEER of 2,940 ± 800 Ω × cm^2^ in monoculture [15], slightly higher than the average TEER reported here although not statistically significant (*p* > 0.05). Maximum TEER of IMR90-4-derived BMECs remained at or above 2,000 Ω × cm^2^ across a wide range of hPSC starting densities. On the other hand, both H9- and DF19-9-11T-derived BMECs demonstrated diminished TEER outside their respective optimum hPSC densities, even using the optimized filter seeding protocol. Thus, while IMR90-4-derived BMECs are capable of generating high TEER values across a wide range of starting densities, it may be particularly important to control the day 0 hPSC starting density in hPSCs lines other than IMR90-4 iPSCs to maximize TEER. For example, while H9 hESCs required an extended length of EC treatment phase of 4 days in order to achieve substantial TEER using Versene colony passage [15], controlling the day 0 hPSC seeding density using the singularized-cell seeding approach reported here allowed H9-generated BMECs to achieve high TEER using the standard 2-day EC media treatment. It may be beneficial to perform a seeding density optimization study such as the one presented here when applying the model to new stem cell lines, centered around 30,000 cells/cm^2^.

Another advantage of employing an optimized day 0 hPSC seeding density is that it facilitates the robust scaling of BMEC differentiations. For example, the IMR90-4 iPSC starting density of 30,000 cells/cm^2^ was used to scale down differentiation from previously reported 6-well culture to 96-well culture without noticeably impacting BBB marker expression, capability for barrier formation, or efflux transporter activity. Importantly, scaling down well size during differentiation allows the ability to perform large-scale screens; for example, a single 6-well of hPSCs can be used to seed >300 96-wells for BMEC differentiation.

## Conclusions

Singularized-cell seeding and subculturing protocols described here suggest that a day 0 seeding density of 30,000 cells/cm^2^ and a Transwell subculturing density of 1 × 10^6^ cells/cm^2^ is sufficient to optimize BMEC differentiation efficiency and purified phenotype. It is expected that these parameters will also enable the efficient extension of the differentiation protocol to other hPSC lines and laboratories.

## Additional file 1

Additional file 1 Supplementary characterization of hPSC-derived BMECs

## Abbreviations

BBB: blood–brain barrier
BCRP: breast cancer resistance protein
BMEC: brain microvascular endothelial cell
CNS: central nervous system
CsA: cyclosporin A
DCFDA: carboxymethyl-2′,7′-dichlorofluorescein diacetate
EC: endothelial cell
iPSC: induced pluripotent stem cell
hPSC: human pluripotent stem cell
hESC: human embryonic stem cell
MRP: multidrug resistance protein
MRP-1: multidrug resistance protein-1
PDS: platelet poor plasma-derived serum
PSC: pluripotent stem cell
RA: retinoic acid
ROCK: Rho-associated protein kinase
TEER: transendothelial electrical resistance
UM: unconditioned media.
