# Supplementary material for: Exploring the effects of cell seeding density on the differentiation of human pluripotent stem cells to brain microvascular endothelial cells
Source: Fluids Barriers CNS. 2015 May 21;12:13. doi: 10.1186/s12987-015-0007-9 (PMC4455681; doi:10.1186/s12987-015-0007-9)
Supplement: Additional file 1 — Supplementary characterization of hPSC-derived BMECs [file 12987_2015_7_MOESM1_ESM.pdf]

Supplementary Information

Exploring the effects of cell seeding density on the differentiation of human pluripotent stem cells to brain microvascular endothelial cells

Hannah K. Wilson, Scott G. Canfield, Michael K. Hjortness, Sean P. Palecek, and Eric V. Shusta

Inventory of Supplementary Information

Table S1

Figure S1

Figure S2

Figure S3

Figure S4

Figure S5

Figure S6

Table S1: Antibodies used for immunofluorescence and flow cytometry experiments.

Antibody	Vendor, clone or product number	Dilution
PECAM-1	Thermo Scientific, RB-10333	1:25
Glut-1	Thermo Scientific, SPM498	1:100
VE-cadherin	Santa Cruz, BV9	1:25
Occludin	Invitrogen, OC-3F10	1:50
Claudin-5	Invitrogen, 4C3C2	1:200
P-gp	Thermo Scientific, F4	1:25
BCRP	Millipore, 5D3	1:50
MRP1	Millipore, QCRL1	1:25
Nestin	Millipore, 10C2	1:500

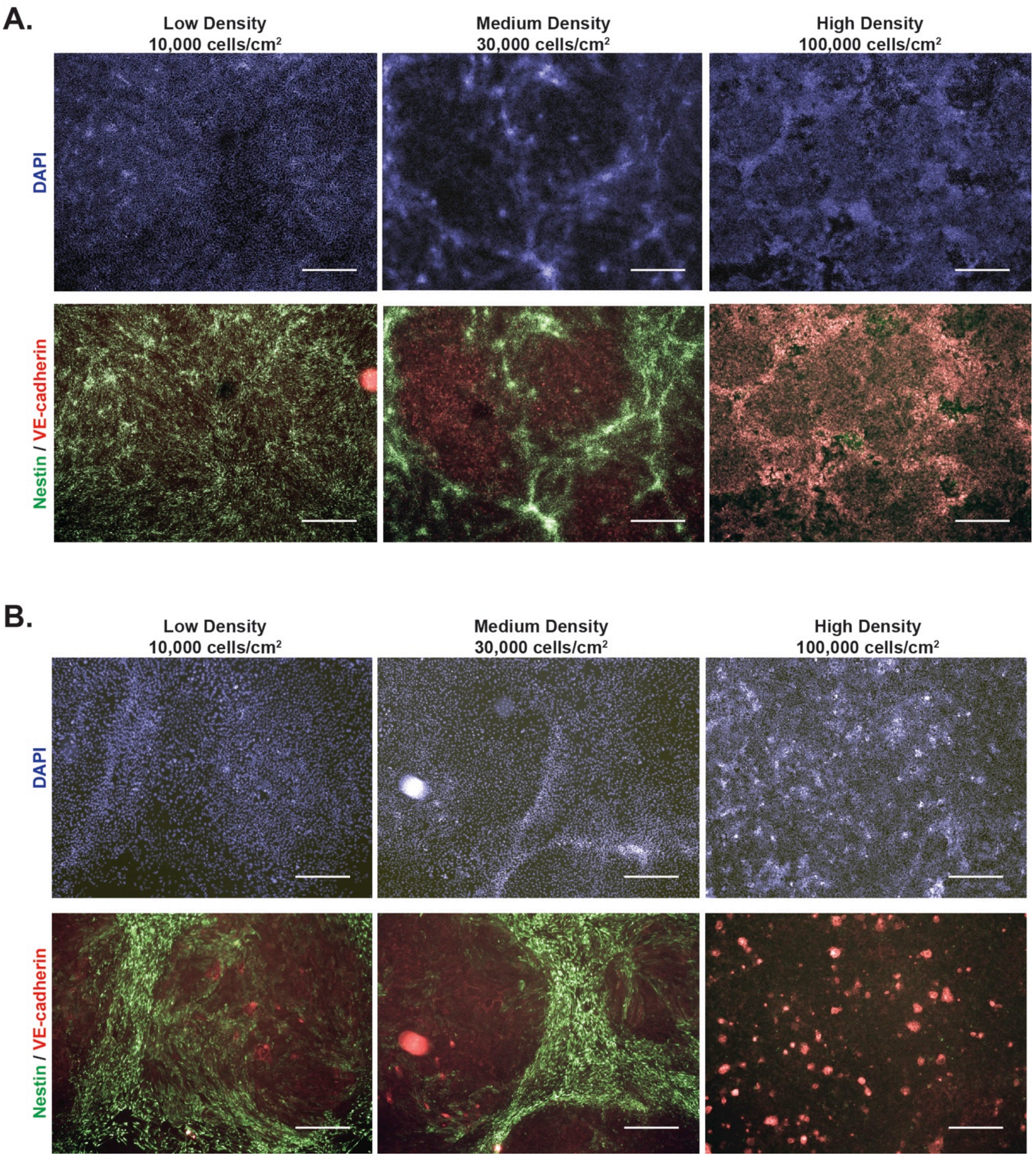

Figure S1: Effect of Day 0 hPSC density on differentiation efficiency and yield in H9 hESCs and DF19-9-11T iPSCs. H9 hESCs (A) and DF19-9-11T iPSCs (B) were differentiated for 8 days, and the mixed cultures were co-stained for VE-cadherin (red) and nestin (green). Recorded densities are the day 0 hPSC starting densities. Scale bars, 500 μm.

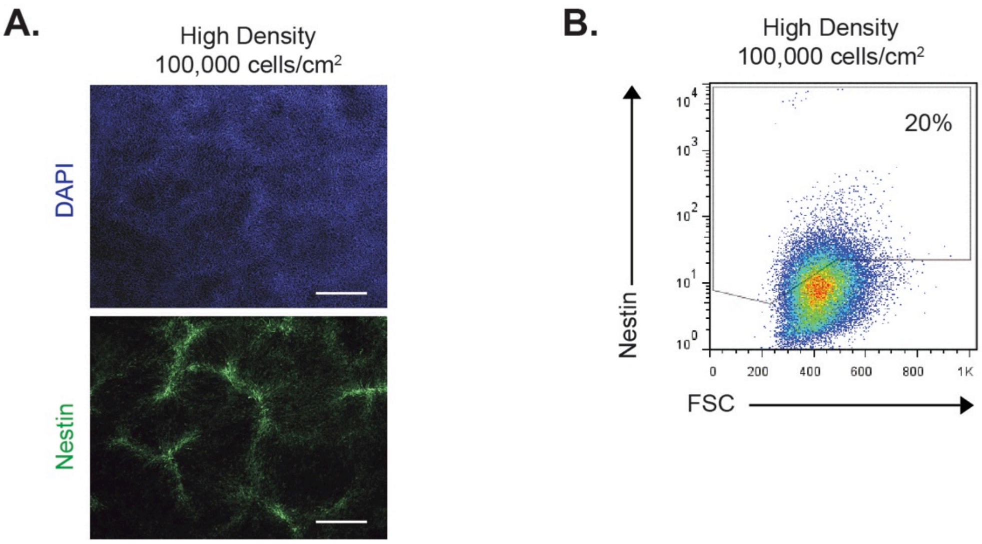

Figure S2: Nestin expression in high density culture at 4 days differentiation. (i) Immunostaining of nestin (green) after 4 days UM treatment. Scale bars, 500 μm. (ii) Flow cytometry dot plot of nestin expression in differentiating cultures after 4 days UM treatment. Gate represents expression above mouse IgG control.

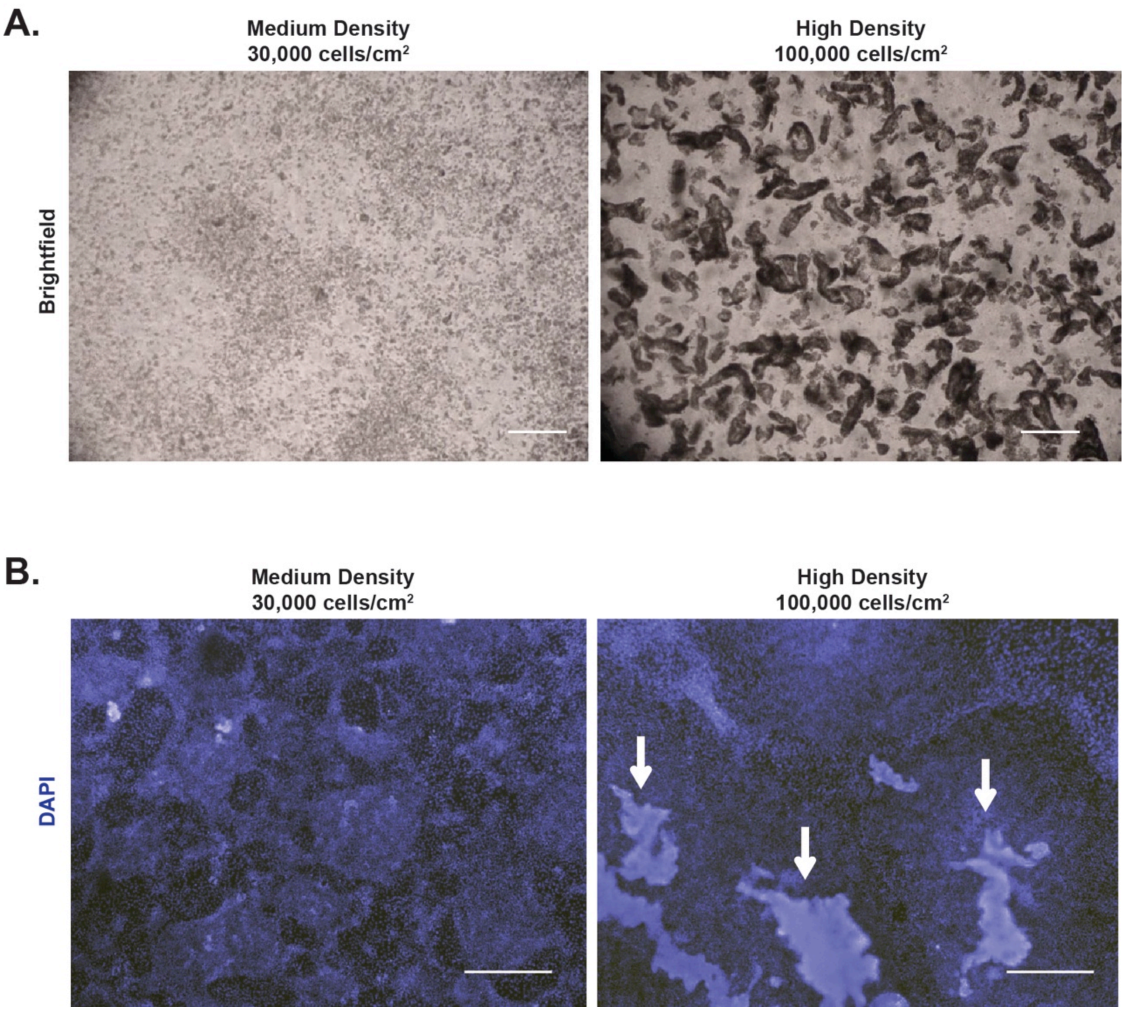

Figure S3: Effect of Versene subculturing on medium- and high-density cultures. (A) Brightfield images of Versene-dissociated IMR90-4-derived BMECs at 8 days differentiation, immediately after dissociation and prior to cell attachment onto collagen/fibronection-coated plates. Scale bars, 500 μm. (B) DAPI staining of Transwells following Versene subculture of IMR90-4-derived BMECs, 2 days after purification. Arrows, clusters of cellular debris. Scale bars, 500 μm.

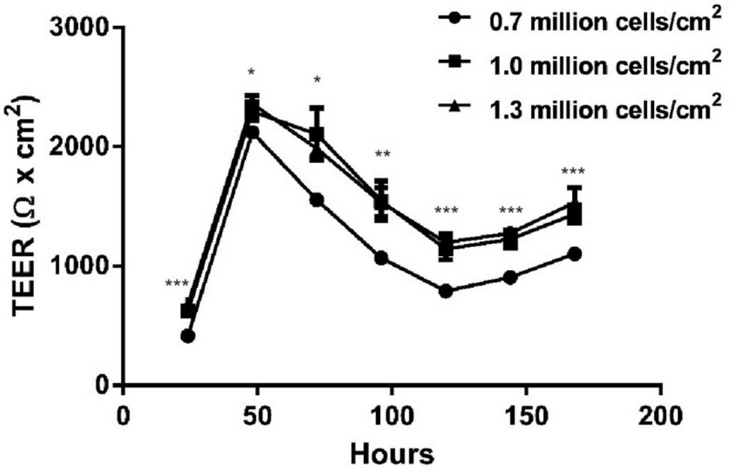

Figure S4: Filter seeding density optimization. IMR90-4 iPSCs were differentiated at a day 0 hPSC starting density of 30,000 cells/cm^2 for 8 days, subcultured using Accutase, and seeded onto 12-well Transwells at a filter seeding density of 0.7, 1.0, or 1.3 million cells/cm^2. Values are mean ± s.d of triplicate Transwells from a single differentiation. The experiment was repeated for an additional 2 independent differentiations to verify trends. Statistical significance between 0.7 and 1.0 million cells/cm^2 was calculated using the student's unpaired t-test (*, $p \leq 0.05$; **, $p \leq 0.01$; ***, $p \leq 0.005$).

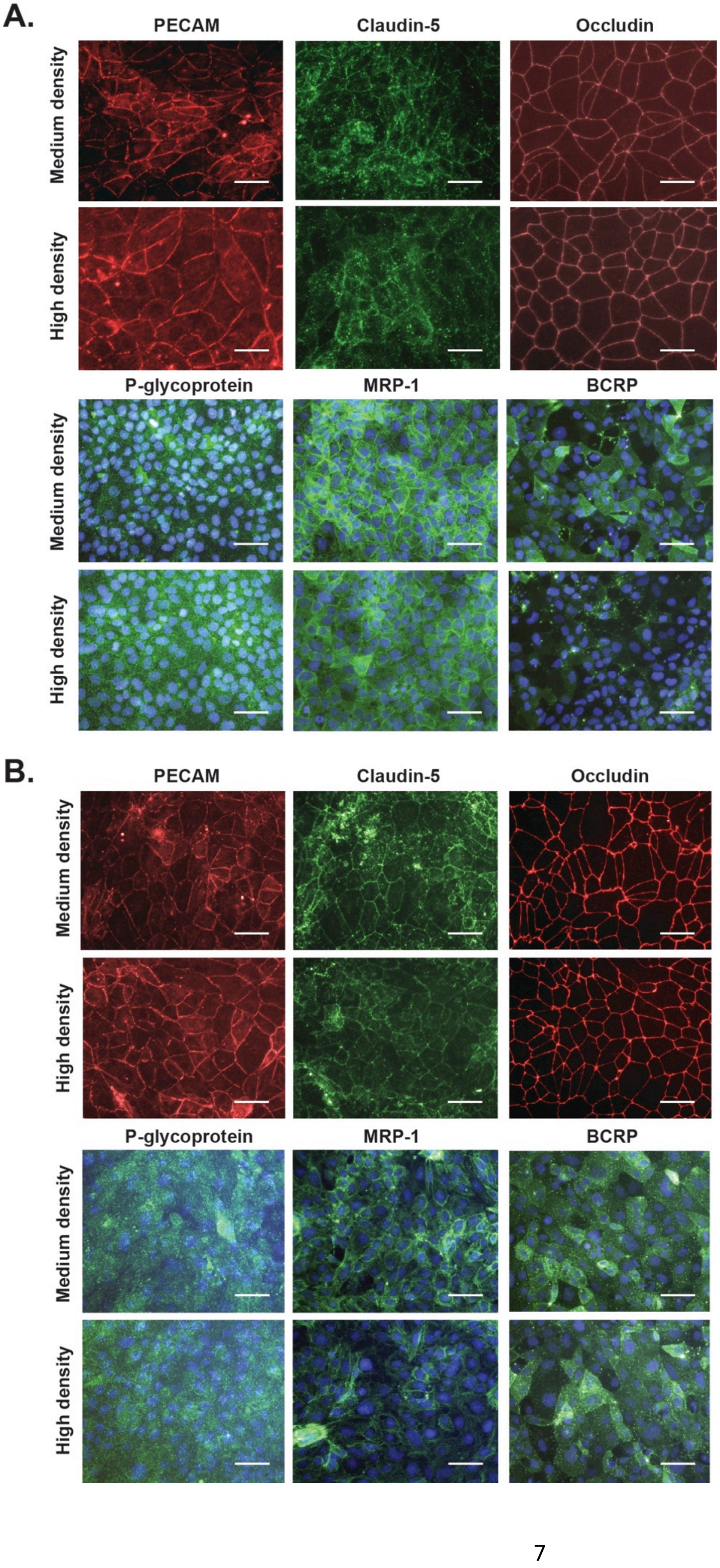
A.
PECAM
Claudin-5
Occludin
Medium density
High density
P-glycoprotein
MRP-1
BCRP
Medium density
High density
B.
PECAM
Claudin-5
Occludin
Medium density
High density
P-glycoprotein
MRP-1
BCRP
Medium density
High density

Figure S5: Expression of BBB markers in H9 hESC- and DF19-9-11T iPSC-derived BMECs. H9 hESCs (A) and DF19-9-11T iPSCs (B) were differentiated at either medium (30,000 cells/cm^2) or high (100,000 cells/cm^2) day 0 hPSC starting density. Immunocytochemistry of purified cultures at day 10 of differentiation reveals expression of PECAM, claudin-5, occludin, P-glycoprotein, MRP-1 and BCRP in both medium- and high-density culture. DAPI overlay is included on MRP-1, BCRP, and P-glycoprotein. Scale bars, 50 μm.

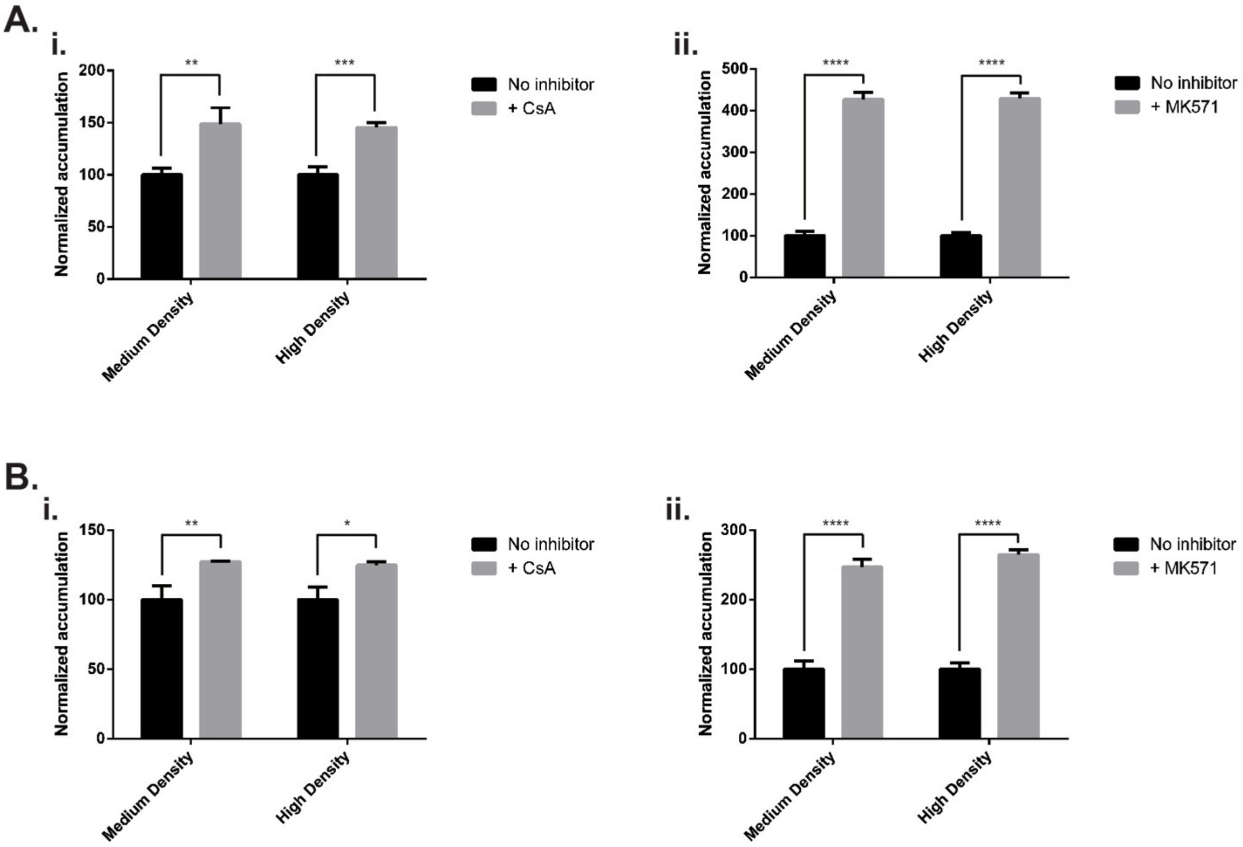

Figure S6: Efflux transporter activity in H9 hESC- and DF19-9-11T iPSC-derived BMECs. H9 hESCs (A) and DF19-9-11T iPSCs (B) were differentiated at either medium (30,000 cells/cm^2) or high (100,000 cells/cm^2) hPSC starting density. Efflux transporter activity was measured on purified cultures at day 10 of differentiation via intracellular accumulation of either rhodamine 123 (Ai and Bi) or DCFDA (Aii and Bii). Inhibitor-treated samples were independently normalized to each respective non-inhibitor-treated control sample. Values are mean ± s.d. of three replicates from a single differentiation, and experiments were repeated for 2 additional independent differentiations to verify reported trends. Statistical significance between each inhibitor condition and its respective non-inhibitor control was calculated using the student's unpaired t-test (*, $p \leq 0.05$; **, $p \leq 0.01$; ***, $p \leq 0.001$; ****, $p \leq 0.0001$).
